# Two new and effective food-extracted immunomodulatory agents exhibit anti-inflammatory response activity in the hACE2 acute lung injury murine model of COVID-19

**DOI:** 10.3389/fimmu.2024.1374541

**Published:** 2024-05-14

**Authors:** Shasha Liu, Baiqiao Wang, Tianran Chen, Hui Wang, Jinbo Liu, Xuan Zhao, Yi Zhang

**Affiliations:** ^1^ Biotherapy Center and Cancer Center, the First Affiliated Hospital of Zhengzhou University, Zhengzhou, China; ^2^ The First Clinical Medical College, Henan University of Chinese Medicine, Zhengzhou, China; ^3^ School of Life Sciences, Zhengzhou University, Zhengzhou, China; ^4^ School of Public Health, Zhengzhou University, Zhengzhou, China; ^5^ Engineering Key Laboratory for Cell Therapy of Henan Province, Zhengzhou, China

**Keywords:** COVID-19, ajoene-enriched garlic extract, sulforaphane, hACE2, anti-inflammatory

## Abstract

**Objective:**

The coronavirus disease 2019 (COVID-19) spread rapidly and claimed millions of lives worldwide. Acute respiratory distress syndrome (ARDS) is the major cause of COVID-19-associated deaths. Due to the limitations of current drugs, developing effective therapeutic options that can be used rapidly and safely in clinics for treating severe acute respiratory syndrome coronavirus-2 (SARS-CoV-2) infections is necessary. This study aims to investigate the effects of two food-extracted immunomodulatory agents, ajoene-enriched garlic extract (AGE) and cruciferous vegetables-extracted sulforaphane (SFN), on anti-inflammatory and immune responses in a SARS-CoV-2 acute lung injury mouse model.

**Methods:**

In this study, we established a mouse model to mimic the SARS-CoV-2 infection acute lung injury model via intratracheal injection of polyinosinic:polycytidylic acid (poly[I:C]) and SARS-CoV-2 recombinant spike protein (SP). After the different agents treatment, lung sections, bronchoalveolar lavage fluid (BALF) and fresh faeces were harvested. Then, H&E staining was used to examine symptoms of interstitial pneumonia. Flow cytometry was used to examine the change of immune cell populations. Multiplex cytokines assay was used to examine the inflammatory cytokines.16S rDNA high-throughput sequencing was used to examine the change of gut microbiome.

**Results:**

Our results showed that AGE and SFN significantly suppressed the symptoms of interstitial pneumonia, effectively inhibited the production of inflammatory cytokines, decreased the percentage of inflammatory cell populations, and elevated T cell populations in the mouse model. Furthermore, we also observed that the gut microbiome of genus *Paramuribaculum* were enriched in the AGE-treated group.

**Conclusion:**

Here, for the first time, we observed that these two novel, safe, and relatively inexpensive immunomodulatory agents exhibited the same effects on anti-inflammatory and immune responses as neutralizing monoclonal antibodies (mAbs) against interleukin 6 receptor (IL-6R), which have been suggested for treating COVID-19 patients. Our results revealed the therapeutic ability of these two immunomodulatory agents in a mouse model of SARS-CoV-2 acute lung injury by promoting anti-inflammatory and immune responses. These results suggest that AGE and SFN are promising candidates for the COVID-19 treatment.

## Introduction

Coronavirus disease 2019 (COVID-19) was a public health emergency of international concern, causing over 632 million confirmed cases and 7 million deaths worldwide. Furthermore, COVID-19 has had a serious socioeconomic impact globally in the last three years (1). Severe acute respiratory syndrome coronavirus-2 (SARS-CoV-2) is the causative agent of COVID-19 and directly binds to the SARS-CoV-2 entry receptor, angiotensin-converting enzyme-2 (ACE2) (2, 3). Infected patients have clinical features ranging from asymptomatic to severe disease. Most critically ill patients deteriorated suddenly in the later stages of the disease and died in a short period of time due to acute respiratory distress syndrome (ARDS), which arises from cytokine release syndrome (CRS) and a complex immune-inflammatory response (4). Global efforts were made to rapidly develop specific anti-SARS-CoV2 antiviral drugs. However, the long process of validating the clinical efficacy and safety limited the drug’s development. Therefore, the current agents proved efficient and safe and required further evaluation in COVID-19 treatment. Emerging evidence has shown that excessive inflammation is a key driver of poor COVID-19 outcomes. The coagulation markers, proinflammatory cytokines, such as interleukin (IL)-6, IL-10, and tumor necrosis factor alpha (TNF‐α), and lymphopenia are associated with the severity of COVID-19 (5–7). Therapeutic immunomodulation is effective in the management of inflammatory diseases. Currently, the immunomodulatory agents include therapies targeting the IL-6/interleukin 6 receptor (IL-6R), TNF-α, and IL-1β pathways (8, 9). These agents have been suggested for treating the advanced stage of COVID-19 by reducing inflammatory responses (10, 11). The clinical outcomes of patients with COVID-19 were improved by these immunomodulatory therapies (4). However, multiple clinical trials of these agents have shown limited benefits (12, 13). Thus, effective, convenient, and inexpensive therapeutic strategies are required to treat SARS-CoV-2 infections.

Natural food products are excellent candidates for use in drug discovery. Medicinal plant extracts have been used to treat various diseases, including viral infection (14–16). Garlic has been consumed by humans for a thousand years as a functional food and a traditional remedy for preventing and treating various diseases since ancient times (17–19). Garlic and its active organosulfur compounds alleviate several viral infections in preclinical and clinical investigations (16). The World Health Organization considers it an essential component of any balanced diet with immunomodulatory properties (20). Ajoene [(E)-(Z)-4,5,9-trithiadodeca-1,6,11-triene-9-oxiade], a major bioactive compound extracted from garlic (21), is synthetically produced by the decomposition of allicin (22, 23). Ajoene has several biological functions, including anti-thrombotic, antioxidant, anti-microbial, anti-viral, anti-cancer, and anti-inflammatory activities, regulating the immune response (24–26). Ajoene exhibits a broad spectrum of *in vitro* and *in vivo* activities against several DNA and RNA viruses (18, 27, 28), including inhibitory activity against human immunodeficiency virus 1 (HIV-1) by blocking HIV-1 virus-cell attachment (29). Sulforaphane (SFN), which is extracted from cruciferous vegetables, including cabbage, broccoli, and Brussels sprouts, induces the activation of nuclear factor-erythroid 2-related factor 2 (NRF2), with acknowledged anti-inflammatory and anti-oxidant properties (30). However, whether ajoene-enriched garlic extract (AGE) and SFN play therapeutic roles in SARS-CoV-2 infection remains unclear.

In this study, we established an effective and biologically safe mouse model that mimicked the pathological processes of ARDS and CRS during SARS-CoV-2 infection (31). We utilized a SARS-CoV-2 acute lung injury mouse model to investigate the role of two immunomodulatory agents, AGE and SFN, in anti-inflammatory and immune response modulation. We examined whether interventions with these two agents could mitigate the inflammatory response and restore the immune system in SARS-CoV-2 *in vivo.* Here, for the first time, we observed that AGE and SFN significantly decreased the level of lung injury, effectively inhibited the production of inflammatory cytokines, decreased the percentage of inflammatory cell populations, and elevated the number of lymphocytes, such as CD4^+^T and CD8^+^T cells, in a SARS-CoV-2 ARDS mouse model. AGE and SFN treatment had the same effect as an antagonist of IL-6R, the neutralizing antibody suggested for treating advanced COVID-19. These results provide a theoretical basis for applying AGE and SFN in the treatment of patients with COVID-19.

## Results

### Poly(I:C) and SP-induced acute lung injury and CRS in humanized angiotensin-converting enzyme-2 mice

**Figure 1:**
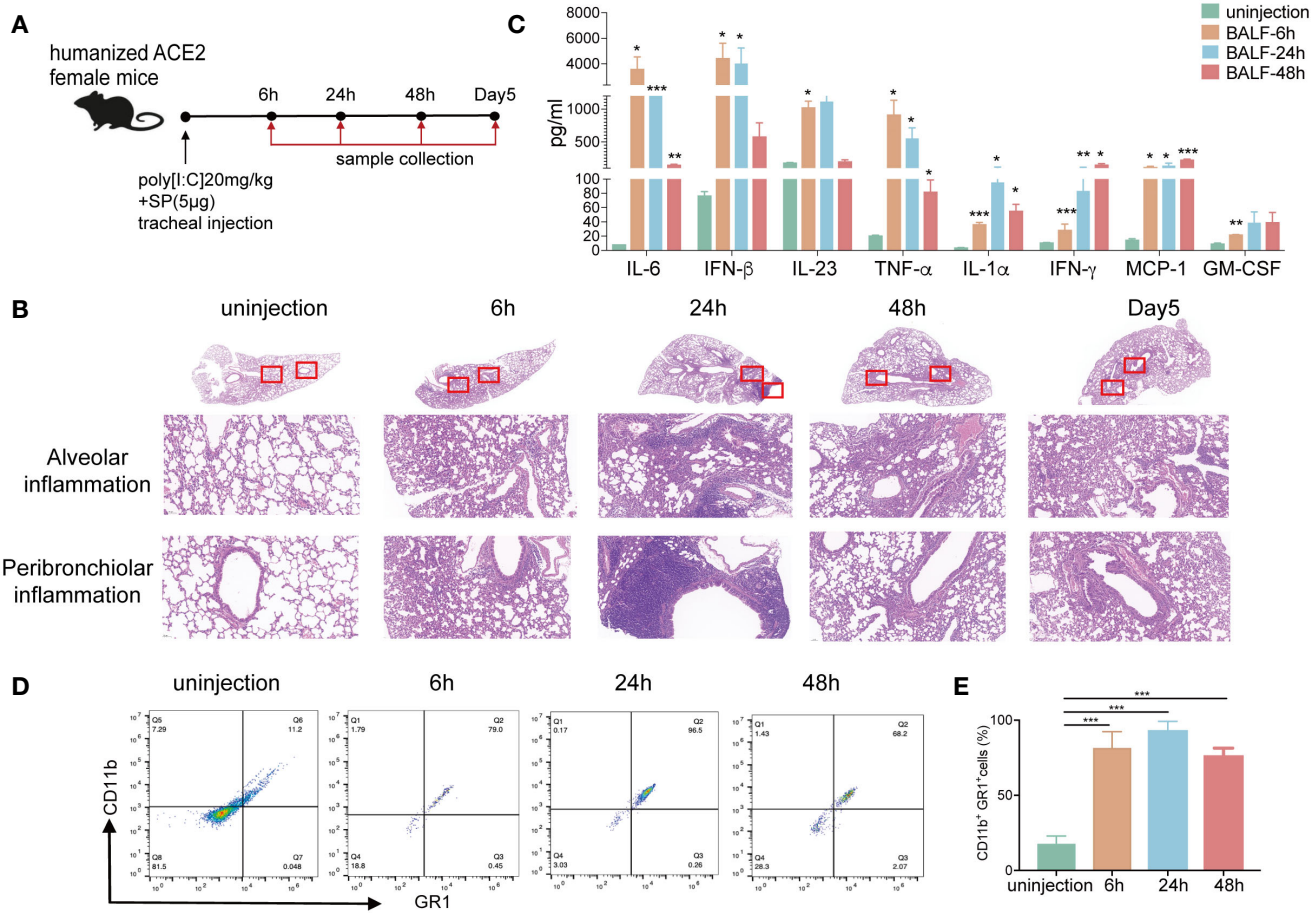
Poly(I:C) and SP-induced acute lung injury and CRS in hACE2 mice. **(A)** Schematic diagram showing the construction of an acute lung injury mouse model via intratracheal injection with 20 mg/kg poly(I:C) and 5 μg SARS-CoV-2 recombinant spike protein (SP). **(B)** Hematoxylin and eosin (HE) staining of histological lung sections of representative normal and injected mice at different time points. Regions of the lung anatomy where alveolar and peribronchiolar inflammation were assessed are highlighted in boxes. Images show low- (up panels) and high-power magnification (down panels) of the same tissue section. **(C)** The inflammation cytokines derived from BALF of the ARDS mouse model at different time points were measured using multiplex cytokines assays. **(D–E)** The percentage of CD11b^+^/GR1^+^ cells derived from the BALF of normal and injected mice was measured using flow cytometry. (n=5). *p <0.05, **p <0.01, and ***p <0.001. ARDS, acute respiratory distress syndrome; BALF, bronchoalveolar lavage fluid; hACE2, humanized angiotensin-converting enzyme-2; poly(I:C), polyinosinic:polycytidylic acid; SARS-CoV-2, severe acute respiratory syndrome coronavirus-2.

To mimic the SARS-CoV-2 infection acute lung injury model, we established a mouse model via intratracheal injection of polyinosinic:polycytidylic acid (poly[I:C]) and SARS-CoV-2 recombinant spike protein (SP) (**Figure 1A**). First, to examine the pathologic changes, we analyzed the results of hematoxylin and eosin (HE)-stained lung sections from the poly[I:C] and SP injected and uninjected mice. The results showed an inflammatory process similar to that previously described for this model after SARS-CoV-2 infection (32, 33). Poly(I:C) and SP injected mice showed increased pulmonary pathology with more alveolar and peribronchiolar inflammation than that in uninjected mice at different time points (**Figure 1B**). Lung inflammation emerged at 6 h and was most severe at 24 h. The lung inflammation gradually declined at 48 h and 5 days postinoculation (dpi). Then, as a measure of lung injury, bronchoalveolar lavage fluid (BALF) derived from injected mice showed a significantly increased level of inflammation cytokines, such as IL-6, interferon (IFN)-β, IL-23, TNF-α, IFN-γ, monocyte chemoattractant protein-1 (MCP1), IL-1β, IL-10, and granulocyte-macrophage colony-stimulating factor (GM-CSF) compared with those in uninjected controls (**Figure 1C**), indicating the formation of CRS. In addition, we observed that the percentage of CD11b^+^GR1^+^neutrophils was markedly elevated in the BALF after injection (**Figures 1D, E**). The accumulation of neutrophils is known to initiate and maintain lung inflammatory responses, including ARDS, and has been demonstrated in SARS-CoV-2 infections (34). We observed that CD3^+^ T, CD4^+^ T, and CD8^+^ T cells were reduced in the BALF and peripheral blood of injected mice, consistent with lymphopenia in patients with COVID-19 (6, 35) (**Supplementary Figures 1**, **2**). These results confirmed the successful construction of an inflammatory mouse model mimicking the pathological characteristics of ARDS and CRS in COVID-19 patients.

### Anti-inflammatory effects of AGE and SFN in a SARS-CoV-2 acute lung injury mouse model

**Figure 2:**
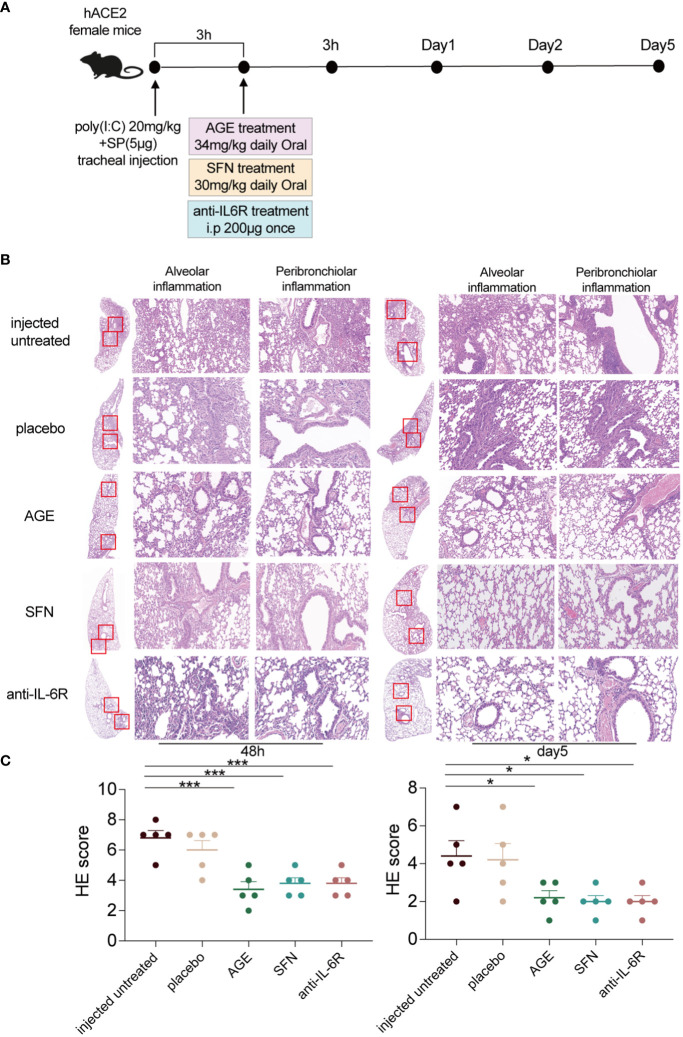
Anti-inflammatory effects of AGE and SFN in the SARS-CoV-2 acute lung injury mouse model. **(A)** Schematic diagram showing the treatment of AGE, SFN, and IL-6R antagonists in a SARS-CoV-2 acute lung injury mouse model. **(B)** HE staining of histological lung sections of the representative injected untreated mice and mice treated with the IL-6R neutralizing antibodies, AGE, and SFN at 48 h and 5 dpi. **(C)** Histopathological severity scoring was evaluated according to the pathological changes. Data from one independent experiment: injected untreated, injected treated. *p <0.05 and ***p <0.001. AGE, ajoene-enriched garlic extract; dpi, days postinoculation; IL-6R, interleukin 6 receptor; HE, hematoxylin and eosin; SARS-CoV-2, severe acute respiratory syndrome coronavirus-2; SFN, sulforaphane.

**Figure 3:**
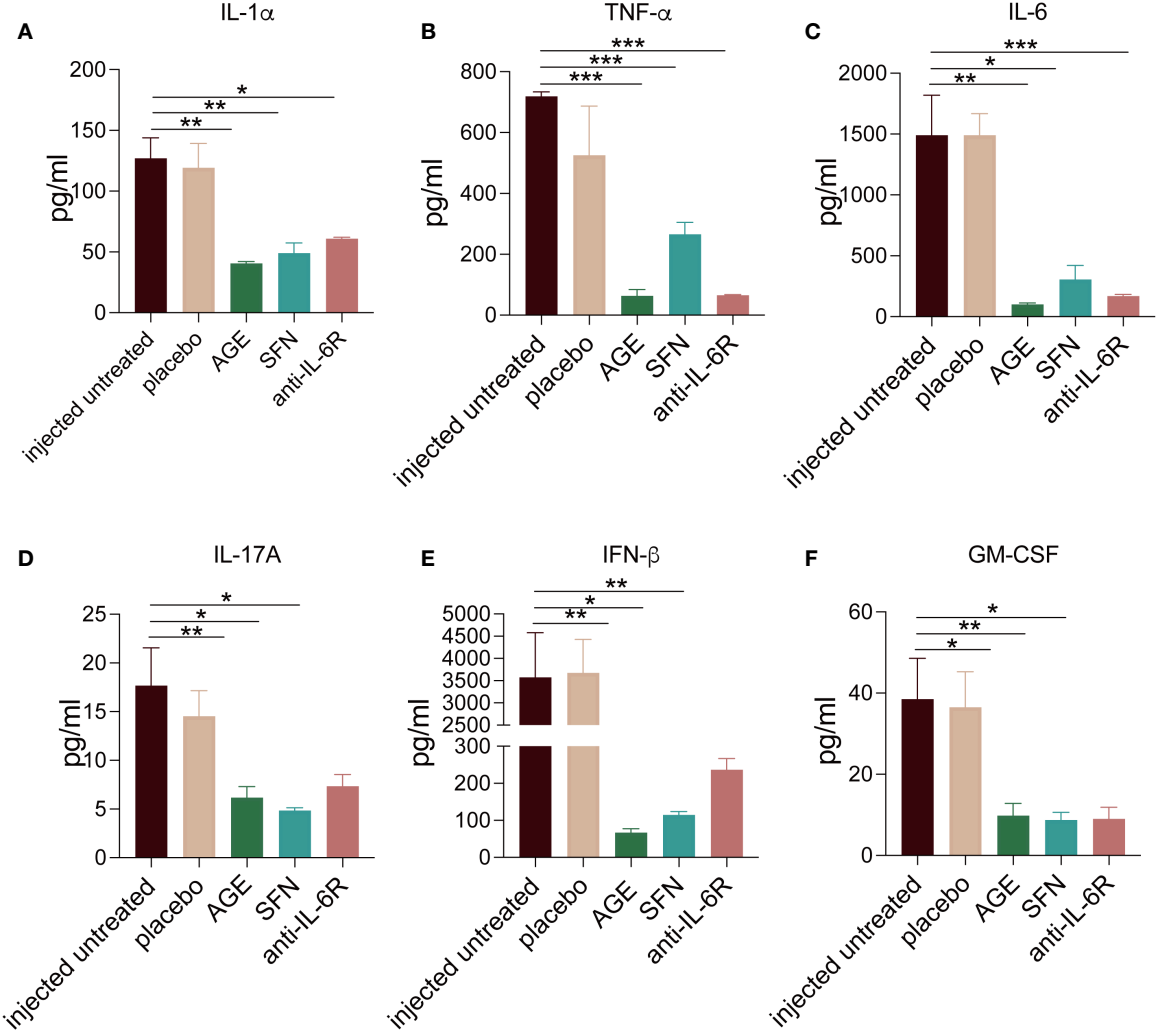
**(A–F)** The inflammatory cytokines changes in the SARS-CoV-2 acute lung injury mouse model BALF after AGE and SFN treatment. After the treatment with AGE, SFN, and IL-6R neutralizing antibodies, the inflammation cytokines from BALF were measured using a multiplex cytokines assay. *p <0.05, **p <0.01, and ***p <0.001. AGE, ajoene-enriched garlic extract; BALF, bronchoalveolar lavage fluid; IL-6R, interleukin 6 receptor; SARS-CoV-2, severe acute respiratory syndrome coronavirus-2; SFN, sulforaphane.

To evaluate the anti-inflammatory ability of AGE and SFN treatment *in vivo*, the SARS-CoV-2 acute lung injury model was used to administer AGE and SFN treatment, and an IL-6R antagonist was used as a positive control (**Figure 2A**). Lung tissues were harvested at different time points for HE staining. Analysis of HE-stained lung sections from these animals showed that the degree of alveolar and peribronchiolar inflammation was attenuated gradually over time after AGE and SFN treatment compared to that in the injected untreated and placebo groups, which showed the same effects as the IL-6R antagonist (**Figure 2B**). Histopathological analysis showed a significant reduction in lung inflammation in AGE- and SFN-treated mice compared to that in untreated controls (**Figure 2C**). Moreover, we examined the levels of inflammatory cytokines in the BALF following treatment with AGE and SFN. The results showed that AGE and SFN treatment had a favorable therapeutic effect in downregulating inflammatory cytokines in BALF, such as IL-1α, TNF-a, IL-6, IL-17A, IFN-β, and GM-CSF (**Figures 3A–F**). Therefore, these results demonstrated that AGE and SFN treatment effectively inhibited acute lung injury and CRS in SARS-CoV-2 injected mouse models by suppressing the degree of pulmonary pathology and inflammatory cytokine production.

### Effects of AGE and SFN treatment on immune response

**Figure 4:**
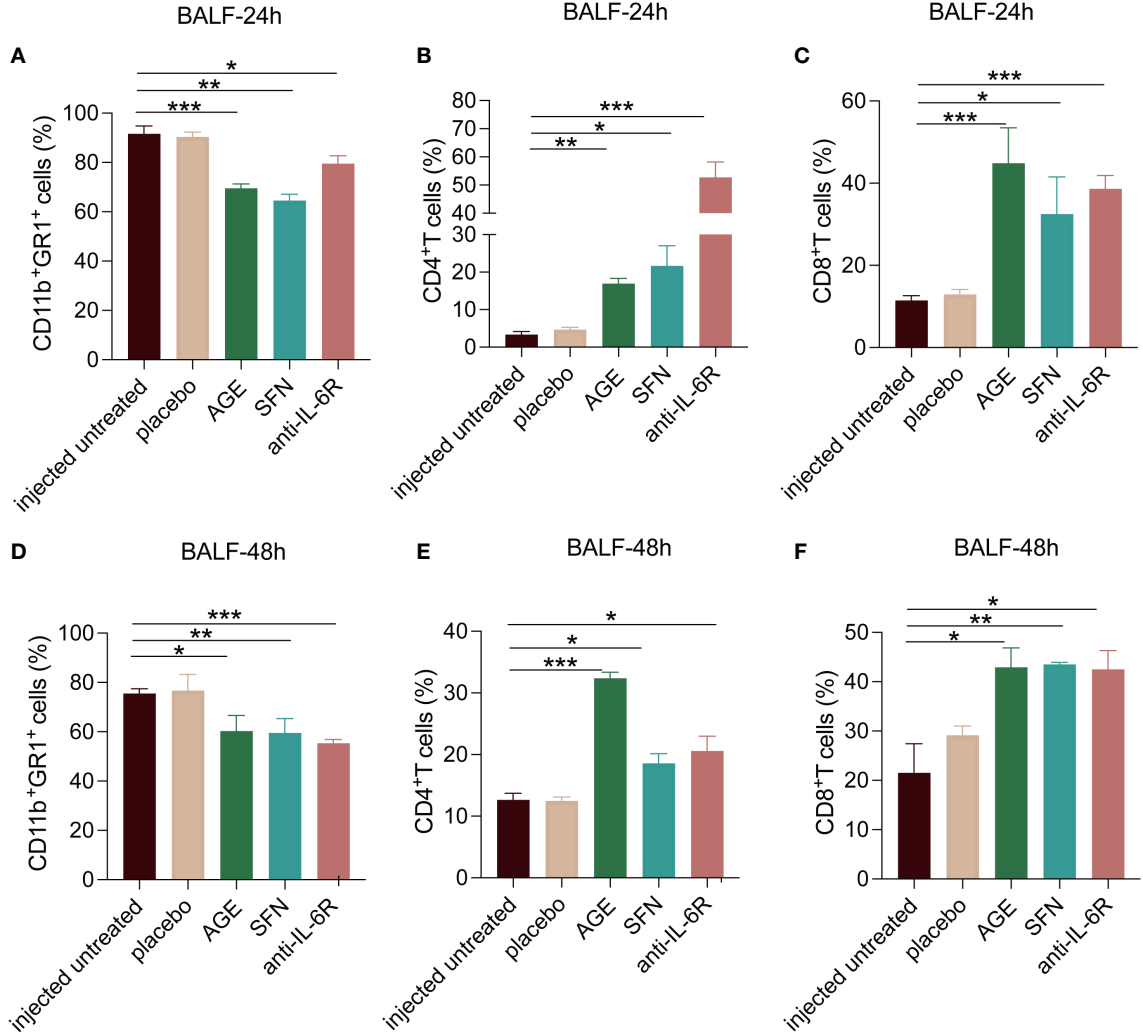
Effects of AGE and SFN treatment on immune response. The percentage of CD11b^+^GR1^+^, CD4^+^T, and CD8^+^T cells in BALF at 24 h **(A–C)** and 48 h **(D–F)** after AGE and SFN treatment was examined using flow cytometry. *p <0.05, **p <0.01, and ***p <0.001. AGE, ajoene-enriched garlic extract; BALF, bronchoalveolar lavage fluid; SFN, sulforaphane.

Several studies have shown that ajoene has many biological functions, including anti-thrombotic, antioxidant, anti-microbial, anti-viral, anti-cancer, and anti-inflammatory activities, and regulates the immune response (24–26). SFN exhibits anti-tumor, anti-inflammatory, and anti-oxidative activities (36–38). Although extensive studies have been conducted, whether and how AGE and SFN modulate the immune response to SARS-CoV-2 infection remains unknown. Therefore, we hypothesized that AGE and SFN may regulate the anti-tumor immune response. To explore the immunomodulatory effects of AGE and SFN, we used flow cytometry to evaluate the changes in the immune response of the SARS-CoV-2 acute lung injury mouse model with different treatments and untreated controls. The results showed that CD11b^+^GR1^+^ neutrophils significantly decreased in BALF after treatment with both AGE and SFN at 24 h and 48 h with the same effects as the IL-6R antagonist (**Figures 4A, D**). We evaluated changes in the composition of other immune cells in BALF. In contrast, AGE and SFN treatment significantly increased the percentage of CD4^+^T and CD8^+^T cells among the total CD45^+^ immune cells in BALF (**Figures 4B, C, E, F**). In summary, these results demonstrated that the immunomodulatory effects of AGE and SFN have a local effect on the immune response in the lungs of a mouse model of SARS-CoV-2 acute lung injury.

### AGE treatment induces intestinal microbiome changes

**Figure 5:**
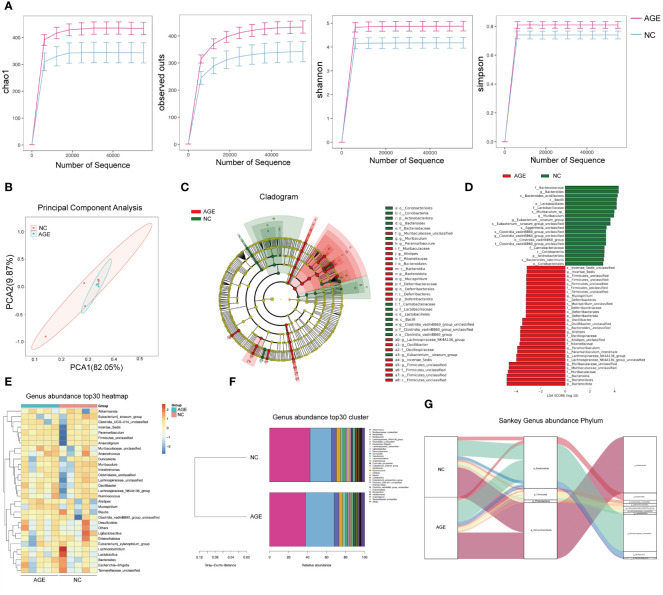
AGE treatment induces intestinal microbiome changes. **(A)** Chao1 indices were observed, and Shannon and Simpson indices were analyzed in the AGE-treated and control groups. **(B)** PCoA of β-diversity using the Bray–Curtis dissimilarity. **(C, D)**. Overall exhibition of effect size (LefSe) analysis using a cladogram in the AGE and NC groups. **(E–G)**. The genus present in the AGE and NC groups was analyzed using a heat map and Sankey diagram. AGE, ajoene-enriched garlic extract; NC, negative control; PCoA, principal coordinate analysis.

There is a growing evidence showing that the gut microbiota, which plays a pivotal role in shaping the inflammation and immune systems, may significantly contribute to the COVID‐19. However, it remains unclear whether AGE treatment has an effect on the gut microbiota in the mouse model of SARS-CoV-2 acute lung injury. Therefore, we also explore the effects of AGE on the gut microbiome of acute lung injury mouse models by amplicon sequencing of 16S rRNA genes. We observed a significant difference between the AGE-treated and control groups in α-diversity, as indicated by the Chao1 indices observed and the Shannon and Simpson indices (**Figure 5A**). In contrast, β-diversity analysis showed no significant differences in the composition and abundance of the gut microbiota (**Figure 5B**). Linear discriminant analysis (LDA) effect size (LEfSe) revealed that the family *Muribaculaceae*, genus *Paramuribaculum*, and species *Paramuribaculum_intestinale* were enriched in the AGE-treated group (**Figures 5C, D**). Subsequent heat map and Sankey diagram analyses confirmed the enrichment of gut bacteria in the genus *Paramuribaculum* (**Figures 5E–G**). These results suggested that *Paramuribaculum* may be crucial for the anti-inflammatory and immunomodulatory effects of AGE.

## Discussion

The SARS-CoV-2 pandemic has substantially impacted global health, leading to a cumulative burden on medical resources, society, and the economy. Although numerous drug discovery efforts have led to the authorization of anti-viral drugs and monoclonal antibodies by the Food and Drug Administration (FDA), inexpensive therapeutics that can be rapidly and conveniently translated into clinical use are urgently required. The immunopathogenesis of COVID-19 involves acute respiratory distress syndrome (ARDS) and organ failure (39). We established a SARS-CoV-2 acute lung injury mouse model to mimic the pathological characteristics of SARS-CoV-2 infection. The mouse model exhibited obvious pathological changes, including alveolar pneumonia, characterized by stronger inflammatory responses, including typical alveolar and peribronchiolar inflammation, increased infiltration of neutrophils in BALF and blood, and serious CRS in the lung tissue, resulting in tissue damage and acute lung injury. A decreased percentage of lymphocytes, such as CD3^+^, CD4^+^, and CD8^+^ T cells, was observed in this model.

The immune system plays a pivotal role in the control and immunopathogenesis of SARS-CoV-2 infection. Lymphopenia is commonly observed in the blood of patients with COVID-19, and the degree of lymphopenia correlates with disease severity (40). Our results are consistent with those of patients with COVID-19 (41). Furthermore, this model provides greater safety and convenience for studying the pathogenic mechanisms and pharmacodynamics of agents against COVID-19 (31).

Garlic has been a common herb consumed as a functional food for thousands of years worldwide. Various garlic extracts have been reported to exhibit therapeutic efficacy against various diseases. Ajoene is a synthetic product of the decomposition of allicin. Growing evidence has demonstrated that allicin and ajoene have effective activities as anti-viral, anti-bacterial, anti-inflammatory, and anti-tumor agents (42, 43). Preclinical studies, both *in vitro* and *in vivo*, have shown that active organosulfur compounds derived from garlic, such as ajoene, allitridin, garlicin, and diallyl sulfide, possess potential anti-viral (28, 44–47), immune-enhancing, and other therapeutic activities (48–50). Ajoene shows a broad spectrum of *in vitro* and *in vivo* activities against zoopathogenic fungi, many yeast strains, zoopathogenic parasites, and several DNA and RNA viruses. Furthermore, in this study, we observed for the first time that AGE could restrain the inflammatory response and recover the immune system, leading to decreased levels of lung injury in SARS-CoV-2 acute lung injury mouse models. Garlic extract reduces neutrophil migration and blocks the biosynthesis of inflammatory cytokines, such as IL-6 and TNF-α (51–54), which supports our results.

The dual anti-viral and anti-inflammatory properties of ajoene against viral infections have been previously described. Ajoene prevents HIV-induced destruction of CD4^+^T cells and enhances cellular immunity *in vitro*. Additionally, it inhibits viral attachment to host cells and the reverse transcriptase of HIV-1 (29). Additionally, a non-organosulfur proteinaceous compound derived from garlic showed anti-viral activity against SARS-CoV by inhibiting early viral attachment and viral activity, which provides evidence for the potential anti-viral activity of ajoene in SARS-CoV-2 (45).

NF-κB activation is crucial for the inflammatory response to multiple viral infections, including COVID-19 (55). Ajoene and other garlic extracts directly inhibit NF-κB activation (56). A previous study showed that garlic extract exerts anti-inflammatory activity by inhibiting lipopolysaccharide (LPS)-induced toll-like receptor-4 dimerization, followed by the suppression of cyclooxygenase (COX)-2 (57). In addition, ajoene can target and covalently modify the signal transducer and activator of transcription 3 (STAT3) and COX-2, leading to an attenuated inflammatory response in macrophages (24). COX-2 catalyzes the synthesis of prostaglandins (PGs), which play a major role in mediating inflammatory responses. Coronaviruses, including SARS-CoV-2, enhance the expression of genes of *COX-1*, *COX-2*, and cytosolic prostaglandin E synthase (*PTGES*) (58). SARS-CoV-2 infection upregulates COX-2 expression *in vivo* in K18-hACE2 mice and *in vitro* in human cells (59). Similarly, COX-1, COX-2, and PTGES were upregulated in peripheral blood mononuclear cells isolated from patients with COVID-19 compared with healthy controls (60, 61). Therefore, these findings support the evidence that ajoene can treat SARS-CoV-2. However, further studies are required to investigate the cellular pathways involved in the antiviral and anti-inflammatory activities of ajoene against SARS-CoV-2.

In recent years, there is a growing evidence show that the gut microbiota, which plays a pivotal role in shaping both the innate and adaptive immune systems, may significantly contribute to the pathogenesis of COVID‐19 (62). The clinical researches found that a notable aspect of COVID‐19 is the prevalence of severe gastrointestinal symptoms reported by approximately 50.5% of affected individuals (63). Furthermore, clinical analyses have demonstrated that individuals with gastrointestinal involvement tend to experience a more severe disease course (64). A clear collection between SARS-CoV-2 infection and notable abnormalities in the gut microbiota, which marked as a decreased bacterial diversity, an increased opportunistic pathogens, a depletion of beneficial commensal organisms, and impaired biosynthesis of essential metabolites was established (65–70). In the humanized ACE2 knock‐in (hACE2‐KI) mouse model, a notable reduction in intra‐individual bacterial richness (α‐diversity) and distinct variations in intergroup microbiota composition (β‐diversity), was observed following SARS-CoV-2 infection (62). In our study, we found that α-diversity and β‐diversity were recovered with the AGE treatment. Garlic has shown the physiological function of balancing intestinal microbiota in other diseases. This is the first report about the effect of AGE on intestinal microbiota in the SARS-CoV-2 acute lung injury mouse model. However, the change of intestinal microbiota following the SARS-CoV-2 infection with the AGE treatment is still unknown and still needs further study.

SFN is an orally accessible dietary phytochemical observed in high amounts in cruciferous vegetables (71). SFN is commercially available at a low cost and has limited side effects (72, 73). SFN has several activities, such as anti-viral, anti-bacterial, anti-inflammatory, and anti-tumor. Ordonez et al. (74) reported that administration of SFN to K18-hACE2 mice before intranasal SARS-CoV2 infection significantly reduced the viral load in the respiratory tract, lung injury, and pulmonary pathology, suggesting that SFN is a potential agent for preventing COVID-19. However, whether SFN is therapeutic in the SARS-CoV-2 ARDS mouse model remains unclear. In this study, we observed that SFN restrained the inflammatory response and restored the immune system, leading to decreased levels of lung injury in a SARS-CoV-2 ARDS mouse model. SFN is an activator of NRF2, which is suppressed in the lung biopsies obtained from patients with COVID-19 (75). Under pathological conditions, reactive oxygen species (ROS) could damage the body through various pathways. When SARS‐CoV‐2 binds to ACE2, the angiotensin II receptor type 1 (AT1R) axis associated with oxidative stress is upregulated, leading to lung and endothelial damage. NRF2 can enhance the ability of cells to scavenge ROS and reduce oxidative damage. The therapeutic effects of NRF2 activation have been demonstrated in animal models of several lung disorders, including respiratory infections and ARDS (76). Sun et al. (30) reported that SFN exerts a significant anti-inflammatory effect on ARDS in rabbits by upregulating NRF2 expression, which is consistent with our results. NF-κB activation and its consequent release of cytokines contribute to the CRS in patients with COVID-19 (77). SFN inhibited NF-κB activation, endowing the anti-inflammatory properties of SFN, which can be another mechanism that explains the beneficial effects of SFN in patients with COVID-19. In addition, NLR family pyrin domain-containing 3 (NLRP3) inflammasome activation is downstream of SARS-CoV2 infection, which is important in inducing inflammatory responses in patients with COVID-19. NLRP3 is inhibited by SFN, independent of the NRF2 pathway. Gasparello et al. (78) reported that downstream cytokines of NLRP3 inflammasome activation, such as IL-6 and IL-8, which are involved in the cytokine storm, were inhibited by SFN. Therefore, targeting the NLPR3 inflammasome with SFN in bronchial cells had beneficial effects. These results are consistent with those of this study.

This study expanded the indications for the clinical application of AGE and SFN and demonstrated that AGE and SFN can inhibit the inflammatory response and recover the immune response in a SARS-CoV-2 acute lung injury model. Given that AGE and SFN are naturally extracted, orally bioavailable, commercially available, and have limited side effects, our results demonstrate that these two immunomodulatory agents can be promising alternative approaches for treating patients with COVID-19 and ARDS.

## Materials and methods

### Animal experiments

Female or male hACE2-All CDS-B6J 6–8-week-old mice were purchased from Cyagen Biosciences (Jiangsu, China). All the mouse experiments were approved by the Ethics Review Commission of the Laboratory Animal Center of Zhengzhou University (Approval number: ZZU-LAC20211015). Mice were housed and bred at the Academy of Medical Sciences, Zhengzhou University Center for Medical Animal Models. The animals were maintained under pathogen-free conditions, and care was provided following the International Association for Assessment and Accreditation of Laboratory Animal Care policies and certifications. The COVID-19 acute lung injury mouse models were constructed according to a previous report (31). Mice were intratracheally administered with the fresh mixture of Poly(I:C)-high molecular weight (HMW, 2.5 mg/mL, InvivoGen, tlrl-picw) and SARS-CoV-2 SP (5 μg, ECD, GeneScript, His&Flag Tag, Z03481). Subsequently, mice were euthanized via cervical dislocation, and samples were collected at different time points for different examinations. The different subgroups of animals received different treatments, including 200 μg neutralizing mAb against IL-6R once intraperitoneal (ip), 32 mg/kg daily AGE diluted in oil, 30 mg/kg daily SFN diluted in oil via oral gavage, and placebo oil. Both neutralizing mAb against IL-6R, AGE and SFN treatment angets were administered at 3h post Poly(I:C) and SARS-CoV-2 SP injection. AGE and SFN were obtained from Puer Qiyun Biotechnology Co., Ltd. (China). Neutralizing mAb against IL-6R (#BE0047) was obtained from Bio X cell (USA).

### BALF sample collection

Mice were euthanized via cervical dislocation to obtain BALF. First, the tracheal lumen was exposed, and the airway was washed with 1 mL of saline solution via a 26G venous indwelling needle inserted into the tracheal lumen. The average total BALF volume was 1 mL per mouse. Then, BALF was centrifuged at 300 g for 10 min at 4°C to separate the supernatants and cells. The supernatants were collected and stored at –80°C for multiplex cytokine assays. The cells were collected for flow cytometry.

### Multiplex cytokines assay

Cytokines or chemokines levels in the sera and BALF of mice were detected using the Mouse LEGENDplex™ Inflammation Panel (13-plex, Biolegend, Cat#740446) following the manufacturer’s instructions. The frozen samples were thawed completely, mixed, and centrifuged to remove particulates before use. The assay was performed using a V-bottom Plate at room temperature (25°C). Samples were read on a 13-laser flow cytometer (CytoFLEX S Flow Cytometer, Beckman), preferably on the same day as the assay. The data were analyzed using BioLegend’s LEGENDplex™ software.

### H&E staining

After euthanasia, the lung tissues were collected and fixed with 10% neutral-buffered formalin. Tissues were dehydrated, embedded in paraffin, sectioned, and mounted on slides. Then, the lung sections were stained with H&E according to standard procedures for examination by digital scanning (Pannoramic MIDI, 3DHISTECH). Histological analysis was subjected by two independent skilled pathologists, in double-blind. Briefly, digital light microscopy scans of the lung were examined implementing a semi-quantitative, 5-point grading scheme (0-within normal limits, 1-mild, 2-moderate, 3-marked, 4-severe) based on what has been previously reported (79). The scoring system considered four different histopathological parameters: (1) perivascular inflammation, (2) bronchial or bronchiolar epithelial degeneration or necrosis, (3) bronchial or bronchiolar inflammation, and (4) alveolar inflammation.

### Flow cytometry

Mice were deeply anesthetized using ketamine and xylazine. Then, the mouse’s chest was opened, the heart was exposed, and a needle was inserted into the right ventricle to collect blood. Blood was collected and treated with red blood cell lysis buffer to collect leukocytes. Leukocytes from the blood and BALF were incubated with fluorescent antibodies (5μL/test) for 30 min on ice in a flow buffer protected from light. Subsequently, the samples were washed with phosphate-buffered saline (PBS) twice and centrifuged to remove the supernatant. The samples were resuspended in 200 μL flow buffer and then ran on a 13-laser CytoFLEX S Flow Cytometer (Beckman). Flow cytometry standard (FCS) files were analyzed using Flowjo v10.6.2 software (BD). The flow cytometry antibodies as follow: FITC anti-mouse CD45 (Biolegend, Cat #103108), Brilliant Violet 510™ anti-mouse CD3 (Biolegend, Cat #100234), APC/Cyanine7 anti-mouse CD8a (Biolegend, Cat #100714), Alexa Fluor® 700 anti-mouse CD4 (Biolegend, Cat #100430), Brilliant Violet 605™ anti-mouse/human CD11b (Biolegend, Cat #101257), Brilliant Violet 650™ anti-mouse Ly-6G/Ly-6C (Gr-1)(Biolegend, Cat #108442),7-AAD Viability Staining Solution (Biolegend, Cat #420403).

### 16S rDNA high-throughput sequencing

Fresh faeces of three mice in each group were collected and stored at –80°C until sequencing. The 16S rDNA high-throughput sequencing was performed by LC-Bio Technology Co. Ltd. (Hangzhou, China). Briefly, DNA from different samples was extracted using trimethylammonium bromide (CTAB) according to the manufacturer’s instructions. The CTAB reagent, designed to extract DNA from trace amounts of the sample, is effective for DNA preparation from most bacteria. Nuclear-free water was used as the blank. The total DNA was eluted in 50 μL of elution buffer and stored at –80°C until measurement using polymerase chain reaction (PCR) performed by the LC-Bio Technology Co., Ltd. (Hang Zhou, Zhejiang Province, China). The 5’ ends of the primers were tagged with specific barcodes per sample and sequenced using universal primers. PCR amplification was performed in a total volume of 25 μL reaction mixture containing 25 ng of template DNA, 12.5 μL PCR Premix, 2.5 μL of each primer, and PCR-grade water to adjust the volume. The PCR conditions to amplify the prokaryotic 16S fragments were initial denaturation at 98°C for 30 s; 32 cycles for denaturation at 98°C for 10 s; annealing at 54°C for 30 s; extension at 72°C for 45 s; and then final extension at 72°C for 10 min. PCR products were confirmed using 2% agarose gel electrophoresis. Throughout the DNA extraction process, ultrapure water instead of a sample solution was used as the negative control to exclude the possibility of false-positive PCR results. The PCR products were purified using AMPure XT beads (Beckman Coulter Genomics, Danvers, MA, USA) and quantified using a Qubit (Invitrogen, USA). Amplicon pools were prepared for sequencing. The size and quantity of the amplicon library were assessed using an Agilent 2100 Bioanalyzer (Agilent, USA) and the Library Quantification Kit for Illumina (Kapa Biosciences, Woburn, MA, USA), respectively. Libraries were sequenced on a NovaSeq PE250 platform. Samples were sequenced on an Illumina NovaSeq platform (LC-Bio) according to the manufacturer’s recommendations.

### Statistics

All data were analyzed using the GraphPad Prism 9.0 software (GraphPad, San Diego, CA, USA). Statistically significant differences were determined using the unpaired Student’s *t*-test and one-way analysis of variance (ANOVA) according to the experimental requirements. *P*-value <0.05 was considered statistically significant, *p<0.05, **p<0.01, and ***p<0.0001.

## Data availability statement

The data presented in the study are deposited in the Genome Sequence Archive in National Genomics Data Center, China National Center for Bioinformation/Beijing Institute of Genomics, Chinese Academy of Sciences that are publicly accessible at https://ngdc.cncb.ac.cn/gsa. The accession number CRA016284.

## Ethics statement

The animal study was approved by Ethics Review Commission of the Laboratory Animal Center of Zhengzhou University. The study was conducted in accordance with the local legislation and institutional requirements.

## Author contributions

SL: Writing – original draft, Writing – review & editing. BW: Writing – original draft, Writing – review & editing. TC: Methodology, Writing – review & editing. HW: Writing – review & editing, Methodology. JL: Writing – review & editing, Methodology. XZ: Writing – original draft, Writing – review & editing. YZ: Writing – original draft, Writing – review & editing.
