# Supplementary figures and images for: Two new and effective food-extracted immunomodulatory agents exhibit anti-inflammatory response activity in the hACE2 acute lung injury murine model of COVID-19
Source: Front Immunol. 2024 May 14;15:1374541. doi: 10.3389/fimmu.2024.1374541 (PMC11130445; doi:10.3389/fimmu.2024.1374541)

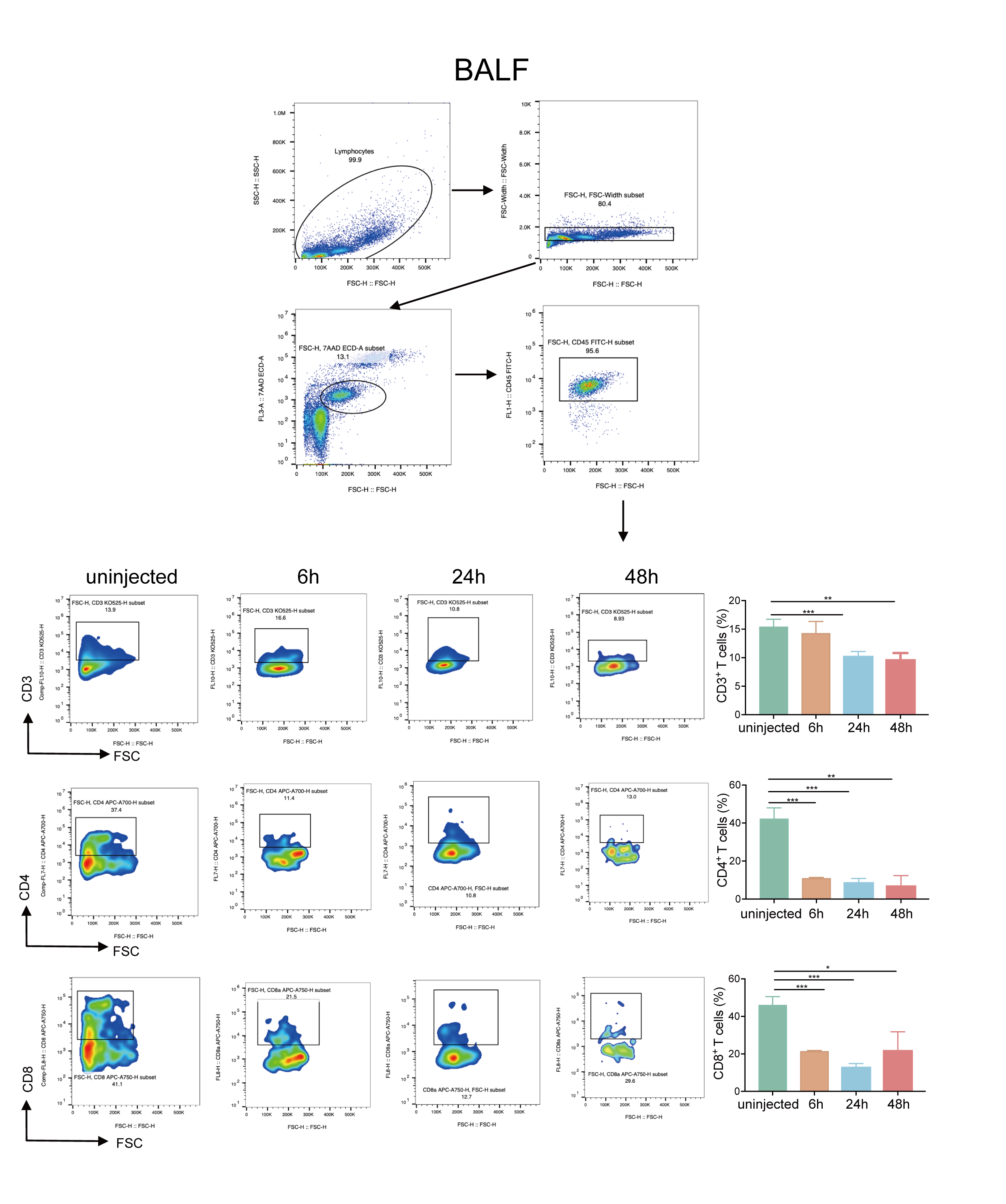

Supplement: Supplementary file 1 [file Image_1.tif]

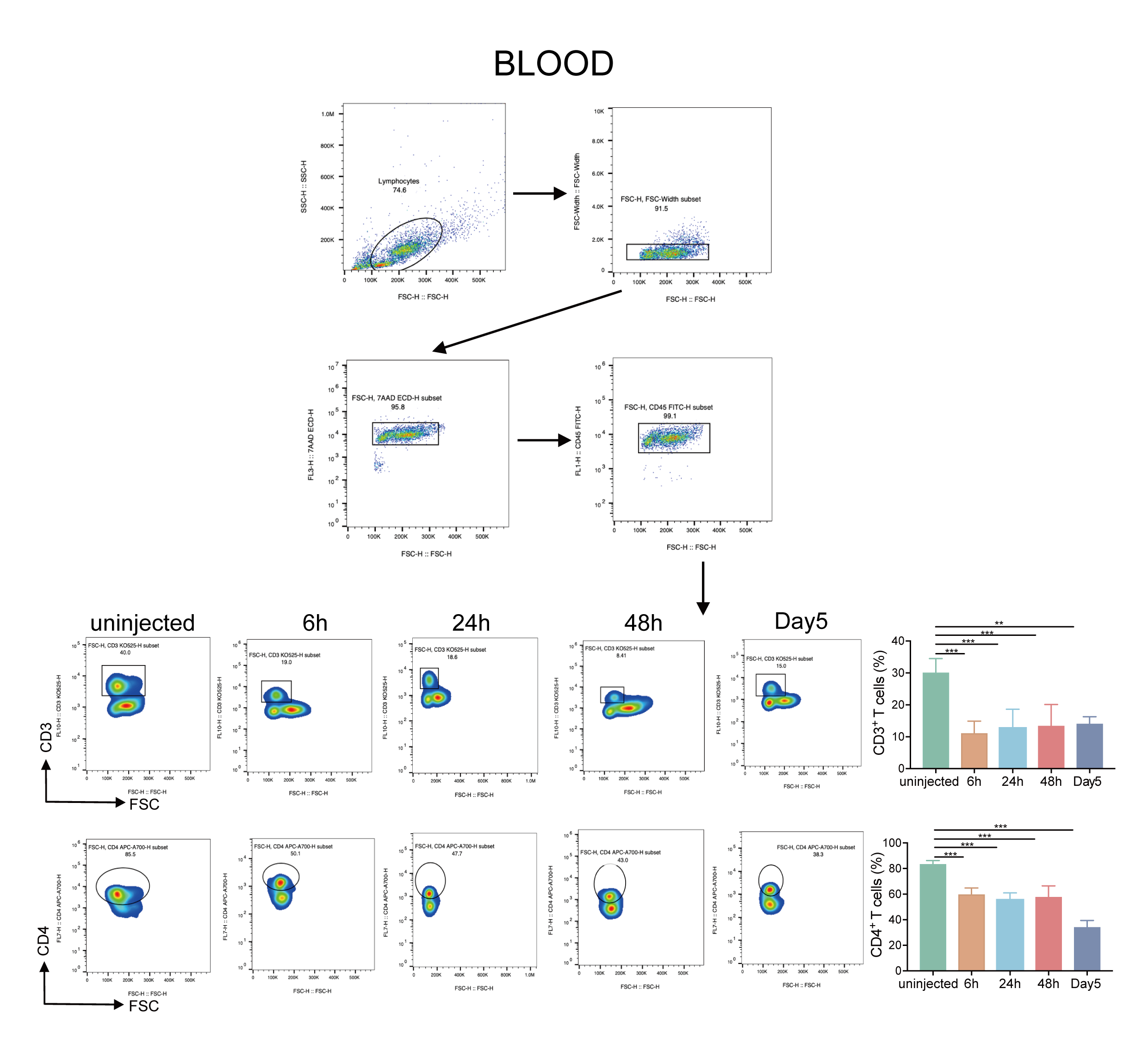

Supplement: Supplementary file 2 [file Image_2.tif]
